# COVID-19, infection fatality rate (IFR) implied by the serology, antibody, testing in New York City

**DOI:** 10.1016/j.idm.2025.10.004

**Published:** 2025-10-28

**Authors:** Linus Wilson

**Affiliations:** Department of Economics & Finance, B.I. Moody III College of Business, University of Louisiana at Lafayette, Moody Hall, Room 253, P.O. Box 43709, Lafayette, LA, 70504, USA

**Keywords:** Antibody, CFR, COVID-19, Death rates, IFR, New York city, SARS-CoV-2, Serology, Seroprevalance

## Abstract

This paper estimates COVID-19 infection fatality rate (IFR) in early 2020 before pharmaceutical interventions were available on a large population in the United States. The better estimates of COVID-19 deaths in New York City and its high COVID-19 infection rate made it ideal to accurately estimate the IFR. Further, we analyze the deaths and infections in New York City to estimate an overall IFR for the United States of 0.86 percent.

## 1 Introduction

The infection fatality rate (IFR) of COVID-19 is one of the most important measures for policymakers considering their response to the pandemic. An infection of COVID-19 is caused by the spread of the novel SARS-CoV-2 virus. The IFR is the ratio of COVID-19 deaths to total infections. Based on data from New York City adjusted for the demographics of the U.S. population we find that the infection fatality rate is 0.86 percent. For males and females in the U.S., we estimate the IFR is 0.98 and 0.74 percent, respectively. The COVID-19 IFR for men over 75 is estimated here as high as 9.1 percent.

Infections were probably grossly undercounted in official case counts. This paper finds from a survey of antibody studies that total infections are on average undercounted by a ratio of 25-to-1. Nevertheless, the range of undercounting of actual to official cases ranges widely from 8-to-1 to 68-to-1 in the serology studies reported on prior to May 2020. Official cases may be undercounted due to testing shortages or asymptomatic spread. The asymptomatic spread was estimated to be over 50 percent in Sutton et al. (2020) and Gudbjartsson et al. (2020). Thus, case fatality rates can grossly overestimate infection fatality rates due to under diagnoses of the COVID-19 infection. Using serology studies by the CDC, Wilson (2022a) estimated that there were four times more U.S. COVID-19 infections than were reported by official case counts by August 13, 2020.

Almasy et al. (2020), found that on April 28, 2020, the U.S. passed 1 million nasal swab (RNA) confirmed infections. Nevertheless, this paper's review of COVID-19 antibody sample studies across the U.S. and in Europe indicate that the real number of infections on April 28, 2020, in the U.S. was probably closer to 25 million or about 7.6 percent of the United States population. In contrast, Wilson (2022a) estimates there were only 19.6 million U.S. COVID-19 infections by August 13, 2020. It is clear that the total number of infections were undercounted greatly throughout 2020 by official case counts.

We believe that the New York City data is some of the best to measure IFR. This is because New York City has a high infection rate and also takes steps to avoid undercounting COVID-19 deaths. Serology, or antibody, studies such as Bendavid et al. (2021) may be vulnerable to a bias towards false positives if the population sampled is not highly infected. Bendavid et al. (2021), which surveyed Santa Clara County, California, and found an approximately 3 percent infection rate on April 3 and 4, 2020. Bendavid et al. (2021), is much more prone to overstate infection rates than New York City with an estimated infection rate of 21 percent on April 22, 2020. Moreover, Katz et al. (2020) find that New York City had the lowest percentage of unaccounted for “excess deaths” of the eight locations surveyed. That is in part because COVID-19 deaths in New York City are not limited to people who died with a positive nasal swab test for the virus. New York City also includes presumed COVID-19 deaths in its official counts. Thus, we believe the IFR calculation here should have greater applicability than ones obtained from other seroprevalence studies reliant on confirmed COVID-19 deaths.

## 2 The infection fatality rate for New York City and the United States

In this section, we will use the summary results from CNBC (2020a) of the New York State serology study on April 22, 2020, with COVID-19 fatality data of NYC Health (2020) to derive the overall and age and gender-specific infection fatality rates (IFRs) for New York City and the United States. To do that we will use population projections from New York City (2013) and the 2010 U.S. Census from Howden and Meyer (2011, pp. 1–15).

On April 22, 2020, there were 10,290 novel coronavirus deaths confirmed in NYC Health (2020). NYC Heath (2020) gives COVID-19 confirmed deaths in five age categories, 0 to 17, 18 to 44, 45 to 64, 65 to 74, and 75+. There were 2 deaths of unknown age which were assigned to the other age categories by the age bracket population weights of all persons, males, and females, respectively from New York City (2013). NYC Health (2020) only breaks down the 10,290 confirmed COVID-19 deaths by age and sex. Maruthappu, and WatkinsNoorWilliamsAliSullivanZeltnerAtun (2020) says the city reported 5121 presumed COVID-19 deaths that were not confirmed by a nasal swab test. The deaths by age were multiplied by the ratio of total confirmed and suspected COVID-19 deaths, 15,411 persons, over confirmed deaths, 10,290 persons. That is, the total deaths in each confirmed age category were increased by the ratio of confirmed and suspected deaths over confirmed COVID-19 deaths, (15,411/10,290) = 1.4977, to avoid undercounting of COVID-19 deaths due to limited testing facilities in 2020.

Katz et al. (2020) uses a Centers for Disease Control (CDC), National Vital Statics System (NVSS) data to estimate “excess deaths” in six states, New York City, and New York State excluding New York City between March 8, 2020, and April 11, 2020. In the seven states not including New York City, the gap, excess deaths minus COVID-19 confirmed deaths, are on average 101 percent of confirmed COVID-19 deaths with a median of 112 percent of COVID-19 deaths. In contrast in New York City where confirmed and presumed COVID-19 deaths are counted, the gap is only 16 percent of confirmed and presumed deaths. Katz et al. (2020) estimated 11,900 excess deaths in New York. 100 % ∗ (11,900–10,261)/10,261 = 16 %. For this reason, we believe that counting confirmed and presumed COVID-19 deaths is the more accurate measure. This also makes the case for calculating IFR from New York City data. In New York City, presumed deaths are counted, and this will avoid underestimation of IFR. One could make the case that our IFR should be 16 perent higher based on Katz et al. (2020)'s excess deaths calculation. Nevertheless, we feel some surge in non-COVID-19 deaths is likely. There may be other deaths caused by the shut-down, medical procedure bans, economic distress and depression, and fear of seeing one's doctor. If people skip cancer screening or treatment and stent procedures for clogged arteries or committed suicide in higher rates, that could add to the excess death tolls without reflecting on the lethality of COVID-19. See Maruthappu, and WatkinsNoorWilliamsAliSullivanZeltnerAtun (2020), Garcia et al. (2020), and Reeves, and StucklerMcKeeGunnellChangBasu (2020) about cancer deaths rising in the Great Recession, the fall in stent procedures during March 2020, and the positive relationship between suicides and unemployment, respectively.

CNBC (2020a) estimated from the serology study that 21.2 percent of New York City residents were infected. Total infected persons were calculated as 1,812,806 based on multiplying the infection rate of 21.2 percent with the 2020 New York City population projection of 8,550,972 from New York City (2013). That is how column (1) of Table 1 was derived.

**Table 1:** COVID-19 Infection fatality rates in New York City by age category and gender.

Age range	(1) IFR for Males and Females	(2) IFR Males Only	(3) IFR Females Only
0 to 17	0.002 %	0.002 %	0.001 %
18 to 44	0.087 %	0.111 %	0.067 %
45 to 64	0.822 %	1.051 %	0.634 %
65 to 74	2.626 %	3.358 %	2.027 %
75+	7.137 %	9.127 %	5.508 %

IFR stands for the infection fatality rate. This is based on infection rates and confirmed and presumed novel coronavirus cases by April 22, 2020, in New York City.

Someone in the 75+ age bracket has a 7.2 percent chance of death if he or she is infected with COVID-19. The chance declines for persons in younger age brackets. Let *IFR*_*t*_ be the population IFR for the age bracket *t* where *t* = 1, 2, 3, 4, and 5 which maps to the 0 to 17, 18 to 44, 45 to 64, 65 to 74, and 75+ age brackets, respectively. Further *w*_*t*_ is the percent of the New York City population in that bracket given by column (1) it Table 2

(1) $$IFR=0.850\%=\sum_{t=1}^{5}w_{t}IFR_{t}$$

**Table 2:** Ages and genders of the 2020 New York City population and the 2010 U.S. population and the infection fatality rates of those groups.

Age range	(1) % of New York City Population	(2) % of New York City Males	(3) % of New York City Females	(4) % of 2010 U.S. Population	(5) % of 2010 U.S. Males	(6) % of 2010 U.S. Females
0 to 17	21.5 %	23.1 %	20.0 %	24.1 %	25.1 %	23.2 %
18 to 44	41.3 %	42.1 %	40.5 %	36.4 %	37.3 %	35.6 %
45 to 64	23.4 %	22.9 %	23.9 %	26.4 %	26.2 %	26.6 %
65 to 74	8.0 %	7.2 %	8.7 %	7.0 %	6.7 %	7.4 %
75+	5.8 %	4.6 %	6.8 %	6.0 %	4.8 %	7.2 %
Total	100.0 %	100.0 %	100.0 %	100.0 %	100.0 %	100.0 %
Population	8,550,972	4,058,814	4,492,158	308,746,241	151,782,029	156,964,212
IFR	0.850 %	0.946 %	0.733 %	0.863 %	0.978 %	0.739 %

IFR stands for infection fatality rate. Population weights for New York City are from the 2020 projections in New York City (2013). U.S. population weights are from the 2010 United States Census in Howden and Meyer (2011, pp. 1–15). Based on its population age distribution, we should expect a higher IFR for the United States than New York City.

The overall IFR for the city can also be found by dividing confirmed and presumed deaths by the infected population, 15,411/1,812,806.

NYC Health (2020) does break out deaths by having one or more additional risk factors. We do not make IFR estimates of comorbidities because there are no good estimates of the subset of the population that have one or multiple of the comorbidities listed in NYC Health (2020). Those “underlying conditions” are diabetes, lung disease, cancer, immunodeficiency, heart disease, hypertension, asthma, kidney disease, GI/liver disease, and obesity. It is simple enough to estimate the percent of Americans who have one of those conditions by age and gender. Unfortunately, the joint distribution of one, two, three, four, five, six, seven, eight, nine, or ten pre-existing conditions is not available. We do know that these health issues are very common. National Center for Health Statistics (2017, p. 221) said in 2013–2014 that 31 percent of U.S. adults over 20-years old suffered from hypertension and 38 percent were obese. Thus, it seems likely that a majority of adult Americans had one or more of those ten risk factors. Persons with no underlying conditions were only 0.59 percent of COVID-19 confirmed deaths in New York City. The rest had known pre-existing conditions, 72.63 percent, or the city had not been able to determine whether or not the persons had pre-existing conditions, 26.77 percent.

NYC Health (2020) does not break out deaths by both age *and* sex. It only breaks out deaths by age *or* sex. To estimate the infection fatality rates by sex, we assumed that COVID-19 was distributed as was found in CNBC (2020b). The New York State serology study found on its April 22, 2020, test that 12 percent of women were COVID-19 positive and 15.9 percent of men were COVID-19 positive. The overall weight of women and men in the New York State antibody study in CNBC (2020b) was 48 percent males and 52 percent females. This is very close to the 2020 break down of men and women in New York City (2013). The New York State serology study in CNBC (2020b) found that 13.9 percent of New Yorkers were COVID-19 positive. Thus, men made up *q*_*m*_ = 0.48∗(0.159)/(0.139) = 54.9 percent of COVID-19 positives. The *q*_*f*_ = 1 – *q*_*m*_ = 45.1 percent. We can only speculate on the discrepancy between men and women sampled. Perhaps women were more careful with hygiene advice or had less exposure in their jobs. We just don't know the reason for the difference. We will assign the infections to men and women based on the weights *q*_*m*_ and *q*_*f*_, respectively.

Thus, 21.2 percent of the projected New York City population or 1,812,806 people are estimated to be infected. Of those, 0.549∗1,812,806 = 995,348. That is our estimate of the males who were infected in New York City. In addition, 0.451∗1,812,806 = 817,458. That is our estimate of total females in New York City who are infected. Dead persons without gender specified are allocated by the relative COVID-19 positive weights for males and females in CNBC (2020b) of 55.1 and 44.9 percent, respectively. After that adjustment for the fifteen gender not reported deaths, NYC Heath (2020) says about 61 percent of confirmed COVID-19 dead by April 22, 2020, were males and 39 percent were females. Using the ratio of confirmed and presumed COVID-19 deaths over confirmed deaths, 15,411/10,290, we extrapolate from that ratio that the COVID-19 deaths consist of about 9419 males and 5992 females. *IFRg* is the overall infection fatality rate by gender, where *g* = *m* or *f*. For males, *IFR*_*m*_ = 0.946 percent = 9419/995,348. For females, the infection fatality rate based on confirmed and presumed deaths is *IFR*_*f*_ = 0.733 percent = 5992/817,458.

The discrepency may be due to underlying biological differences between men and women. Arias et al. (2021) shows that female life expectancy is higher than that of males at every age category. That implies males have higher hazard rates of death overall. Ferguson et al. (2020), Verity et al. (2020), Feng et al., and. Wilson (2022b) all find a large discrepency between the mortality rate of COVID-19 between males and females. Males are much more likely to die from the infection than women. Besides biologial factors women's occupations may have exposed them to lower viral loads from COVID-19 and physical injury throughout their careers. In 2019, females (versus males) were less likely to be in the labor force (57 versus 69 percent); more likely to be in less physically demanding service sector careers (93 versus 69 percent); and more likely in the workforcce to hold a bachelor's degree or higher (45 versus 38 percent) according to U.S. Bureau of Labors Statistics (2021).

Let *w*_*t,g*_ equal the percent of the population in the age bracket, *t*, and gender, *g*. The age-based IFRs by gender and age are in equation (2):

(2) $$IFR_{t,g}=\frac{IFR_{g}\left(IFR_{t}\right)}{\sum_{t=1}^{5}w_{t,g}IFR_{t}}$$

It is easy to verify that age and gender IFRs from equation (2) must sum to the following:

(3) $$IFR_{g}=\sum_{t=1}^{5}w_{t,g}IFR_{t,g}$$

Equation (2) is used to calculate columns (2) and (3) of Table (1) from the population weights in columns (2) and (3) of Table 2. To calculate the overall, male, and female IFRs for the U.S. population we use the *IFR*_*t*_ and *IFR*_*t,g*_ for each age or age and gender grouping in Table 1 columns (1), (2), and (3) and the weights of the overall, male, and female population by age given in columns (4), (5), and (6) in Table 2. Let us denote those population weights from the 2010 U.S. Census in Howden and Meyer (2011, pp. 1–15) as *u*_*t*_ for the age category weights for both males and females combined. *u*_*t,g*_ stands for the weights for males and females separately by age. Let *IFR* and *IFR*_*g*_ denote combined and male or female IFRs for the U.S. population, respectively. They are calculated below:

(4) $${\underline{IFR}}_{g}=\sum_{t=1}^{5}u_{t,g}IFR_{t,g}$$

(5) $$\underline{IFR}=\sum_{t=1}^{5}u_{t}IFR_{t}$$

The overall IFR for the U.S. population is given in the last row of Table 2 column (4) as 0.86 percent, using equation (5). The overall male and female IFRs for the U.S. population are estimated at 0.978 percent and 0.739 percent, respectively using equation (5).

The overall estimate for the U.S. population 0.86 percent is within the 95 percent confidence interval of China IFRs of 0.39–1.33 percent with a point estimate of 0.66 percent from Verity et al. (2020). Wilson (2022a) uses nationwide CDC serology surveys beginning in July 2020, to find the IFR for the U.S. population was 0.85 on average in 2020, with a 95 percent confidence interval of 0.895 percent to 0.806 percent. Since Verity et al. (2020)'s IFR was used in Eichenbaum et al. (2021) then, that should be in line Eichenbaum et al. (2021)'s expected scenarios. Brazeau et al. (2022) found that for high-income countries the serology studies indicated that the early strains of COVID-19 in 2020 had a IFR of 1.1 percent with a 95 percent confidence interval of 0.75–1.72 percent in line with the estimate here.

Nevertheless, the age-based IFRs do differ from Ferguson et al. (2020) because the age categories are different. Further, Ferguson et al. (2020) based its age and age and gender categories on the case fatality rates CFRs in Wuhan, China from Feng et al.. Thus, the age and age-and-gender-based IFRs here are preferable to in Ferguson et al. (2020) if someone is predicting COVID-19 IFRs for the U.S. or other more developed nations’ populations. The World Bank, for example, said the life expectancy at birth in the U.S. was two years higher than in China near the outbreak of COVID-19.

## 3 Serology studies reported prior to April 24, 2020

Early on in the pandemic serology studies were very rare and they implied that the IFR of COVID-19 was lower than the data in New York City suggested. The New York State antibody test is not the only one that had been conducted according to Kekatos (2020). Unfortunately, with the exception of Bendavid et al. (2020), we were unable to obtain accompanying working papers with the many antibody test studies listed in Table 3 prior to May 1, 2020. Table 3 is compiled from the data we could find from mostly news reports or summaries. The sources for Table 3 are Bendavid et al. (2020), Kekatos (2020), CNBC (2020a), New York Governor’s Office (2020), Saltzman (2020), Streeck et al., 2020a, Streeck et al., 2020b, and Van Dissel (2020).

**Table 3:** Serology studies, infection rates, case undercounting, and infection fatality rates.

Location of Serology Sample	Est. % of Pop. Infected	Multiple of Official Cases	Local IFR
Santa Clara County, California, USA	3 %	68	0.16 %
Miami-Dade County, Florida, USA	6 %	15	0.17 %
Uusima, Finland	3 %	29	0.19 %
Los Angeles County, California, USA	4 %	41	0.21 %
Chelsea, Massachusettes, USA	32 %	18	0.31 %
Gangelt, Germany	15 %	8	0.37 %
New York, USA	14 %	10	0.50 %
Neatherlands	3 %	20	0.63 %
New York City, New York, USA	21 %	13	0.85 %
**Average**	**11 %**	**25**	**0.38 %**
**Median**	**6 %**	**18**	**0.31 %**

This is based on the reporting of results seroprevalence results in Bendavid et al. (2020), Kekatos (2020), CNBC (2020a), New York Governor’s Office (2020), Saltzman (2020), Streeck, Hartmann, et al. (2020), and Van Dissel (2020). The New York City estimates are from this paper. Streeck, Schulte, et al. (2020) is the published version of Streeck et al. (2020a). That study published after our cut-off date estimated the IFR at 0.36 percent. Bendavid et al. (2021) is the peer-reviewed version of Bendavid et al. (2020). It also was published after our cut-off date and estimated the local IFR at 0.17 percent. The multiple of official cases are the estimated number of infections in the community tested divided by the official case counts reported by various sources. Local IFR is the infection fatality rate that the sources quoted or the official deaths divided by the estimated number of infected individuals. For New York City, we counted test-confirmed and presumed COVID-19 deaths. New York State in CNBC (2020a) counted only confirmed COVID-19 deaths. The New York City IFR for confirmed only cases is its local IFR divided by 1.497 because presumed deaths from Maruthappu, and WatkinsNoorWilliamsAliSullivanZeltnerAtun (2020) were 49.7 percent of COVID-19 confirmed deaths in New York City.

The studies that estimate a range or point estimates of COVID-19 seroprevalance, which are in the low single digits, may have large false positives problems. All the COVID-19 antibody tests available have specificities that may have not been decisively proven at the time of writing. One minus the specificity is the expected false positive rate. If the specificity of the serology test is 98 percent, it is reasonable to expect 2 percent of the positives are false positives. With a low virus spread, false negatives, one minus the sensitivity, should not be a big issue. Therefore, if the midpoint of the range of COVID-19 positives is 3 percent, then the spread of the infection may be overstated by a factor of 3 on average. True positives should be approximately 3 %–2 % = 1 %. (Roughly, 1 % ∗ 3 = 3 %). Nevertheless, if the virus spread is 20 percent and sensitivity and specificity, are 100 percent and 98 percent, respectively, then false positives should be only 0.8 (1 - 0.98) = 1.6 percent. Thus, the resulting estimated infection rate would be 21.6 percent or just 108 percent of the “true” spread of the infection, 20 percent.

For this reason, we probably should put greater weight on the serology studies of very infected populations. That would mean that the estimates of IFR from New York State, New York City, Chelsea, Massachusetts, and Gangelt, Germany are likely to be less prone to an upward bias in the infected population and a downward bias in IFR estimates. All these locations have estimated double-digit COVID-19 infection rates. Indeed, for the highly infected locations like New York City, the test specificity and sensitivity may bias the results upward or downward. Nevertheless, those biases will be relatively small compared to less infected regions.

Random, population-weighted samples are more prone to false positives when people are tested regardless of symptoms, because our Bayesian prior is that they are probably not infected. Thus, our posterior after a positive test result is far less than certain that they have, in fact, been infected. Our priors will be higher in hard-hit regions like Chelsea, Massachusetts, New York, City, Ganglet, Germany, and New Orleans, Louisiana where hospital beds have filled up and COVID-19 positive deaths have mounted.

We can debate the selection biases inherent in different sampling methods. Bendavid et al. (2020) used targeted Facebook adds and reweighed the results by demographics. They tested 3330 Santa Clara County residents over two days. New York State in CNBC (2020a) approached about 3000 people in supermarkets and big-box stores. The Chelsea, Massachusetts study approached about 200 people on a street corner as described in Saltzman (2020). Van Dissel (2020) describes 4198 people giving blood samples over many days.

For example, while presenting the New York State results in CNBC (2020a), Governor Andrew Cuomo argued that people were shopping during the day and thus likely not “essential workers” with higher exposure to the virus, and they were probably not experiencing symptoms. Thus, the infection rate in New York City and New York State may be biased downward by the under-sampling of essential workers. Thus, our IFR could be overstated by that possible selection bias.

The average IFR of the studies is 0.38 percent with an average multiple of confirmed cases to actual cases of 25-to-1. Thus, on average, we would expect official case counts to be only 4 percent of actual infections of COVID-19. Duster (2020) reported early on April 26, 2020, that the U.S. had 939,000 COVID-19 cases as confirmed by swab test. Table 3 indicates that the real number of COVID-19 infections in the U.S. is likely 23.5 million or about seven percent of the U.S. population.

Contrast this with Sorensen et al. (2022) which estimated that the IFR in the U.S. was 1.280 percent with a 95 percent confidence interval of 0.771–1.877 percent prior to April 15, 2022, using seroprevalance surveys which were not reported in Kekatos (2020). In addition, Meyerowitz-Katz and Merone (2020) found that by July 2020, the average preprint or published article estimated the COVID-19 IFR at 0.68 percent, which is lower than this study predicts, but higher than the IFR implied by the early serology studies, announcing results by May 2020. Meyerowitz-Katz and Merone (2020) did not adjust the IFRs estimated for U.S. population demographics as this study did for the New York City data.

## 4 Conclusion

The COVID-19 pandemic had a lot of uncertainty about the ratio of deaths to total infections in its early strains before pharmaceutical interventions were available. That confounded the calculation of how deadly the novel coronavirus was and the societal costs that should be incurred to slow its spread. The serology sampling in New York City and elsewhere makes estimates of infections more reliable. We analyze the data from New York City in-depth to estimate that the IFR for all ages and genders in New York City was 0.85 percent. New York City is a preferable location to estimate IFR because it had one of the highest infection rates in the world. Thus, random sampling is less prone to an upward bias in false positives. In addition, New York City's official counts are less likely to understate deaths than in other locations in the United States. We find that the infection fatality rates from New York vary a great deal by age and gender. Females ages 0 to 17 can expect infection fatality rates of 0.001 percent while males of age 75 and over can expect infection fatality rates of 9.127 percent.

## Conflicts of interest

I have no conflicts of interest for manuscript number IDM-D-24-00239.
